# 2-Ethyl­piperidinium chloride

**DOI:** 10.1107/S1600536811036804

**Published:** 2011-09-17

**Authors:** Mohammad T. M. Al-Dajani, Jamal Talaat, Abdusalam Salhin, Madhukar Hemamalini, Hoong-Kun Fun

**Affiliations:** a School of Pharmaceutical Sciences, Universiti Sains Malaysia, 11800 USM, Penang, Malaysia; b Virginia Commonwealth University, Chemistry School, USA; c School of Chemical Sciences, Universiti Sains Malaysia, 11800 USM, Penang, Malaysia; d X-ray Crystallography Unit, School of Physics, Universiti Sains Malaysia, 11800 USM, Penang, Malaysia

## Abstract

In the title molecular salt, C_7_H_16_N^+^·Cl^−^, the piperidinium ring adopts a chair conformation. In the crystal, the two components are connected by N—H⋯Cl and C—H⋯Cl hydrogen bonds, forming a supra­molecular double-chain structure along the *c* axis.

## Related literature

For biological applications of piperidine, see: Waelbroeck *et al.* (1992 ▶); El Hadri *et al.* (1995 ▶). For puckering parameters, see: Cremer & Pople (1975 ▶). For the stability of the temperature controller used in the data collection, see: Cosier & Glazer (1986 ▶).
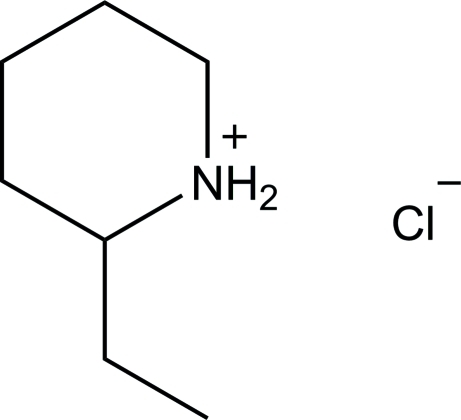


## Experimental

### 

#### Crystal data

C_7_H_16_N^+^·Cl^−^
*M* *_r_* = 149.66
Orthorhombic, 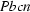

*a* = 24.2052 (6) Å
*b* = 9.7594 (3) Å
*c* = 7.2764 (2) Å
*V* = 1718.89 (8) Å^3^
*Z* = 8
Mo *K*α radiation
μ = 0.37 mm^−1^
*T* = 100 K
0.72 × 0.27 × 0.15 mm

#### Data collection

Bruker SMART APEXII CCD area-detector diffractometer
Absorption correction: multi-scan (*SADABS*; Bruker, 2009 ▶) *T*_min_ = 0.778, *T*_max_ = 0.948
50389 measured reflections
4453 independent reflections
3438 reflections with *I* > 2σ(*I*)
*R*_int_ = 0.045

#### Refinement

*R*[*F* ^2^ > 2σ(*F* ^2^)] = 0.040
*wR*(*F* ^2^) = 0.088
*S* = 1.07
4453 reflections
91 parameters
H atoms treated by a mixture of independent and constrained refinement
Δρ_max_ = 0.34 e Å^−3^
Δρ_min_ = −0.27 e Å^−3^

### 

Data collection: *APEX2* (Bruker, 2009 ▶); cell refinement: *SAINT* (Bruker, 2009 ▶); data reduction: *SAINT*; program(s) used to solve structure: *SHELXTL* (Sheldrick, 2008 ▶); program(s) used to refine structure: *SHELXTL*; molecular graphics: *SHELXTL*; software used to prepare material for publication: *SHELXTL* and *PLATON* (Spek, 2009 ▶).

## Supplementary Material

Crystal structure: contains datablock(s) global, I. DOI: 10.1107/S1600536811036804/is2773sup1.cif

Structure factors: contains datablock(s) I. DOI: 10.1107/S1600536811036804/is2773Isup2.hkl

Supplementary material file. DOI: 10.1107/S1600536811036804/is2773Isup3.cml

Additional supplementary materials:  crystallographic information; 3D view; checkCIF report

## Figures and Tables

**Table 1:** Hydrogen-bond geometry (Å, °)

*D*—H⋯*A*	*D*—H	H⋯*A*	*D*⋯*A*	*D*—H⋯*A*
N1—H1*NA*⋯Cl1^i^	0.886 (14)	2.220 (14)	3.1054 (7)	176.9 (11)
N1—H2*NA*⋯Cl1	0.899 (15)	2.217 (15)	3.1149 (7)	177.8 (12)
C1—H1*A*⋯Cl1^ii^	0.99	2.80	3.6121 (8)	139

Symmetry codes: (i) 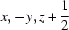; (ii) 
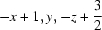
.

**Fig. 1.**
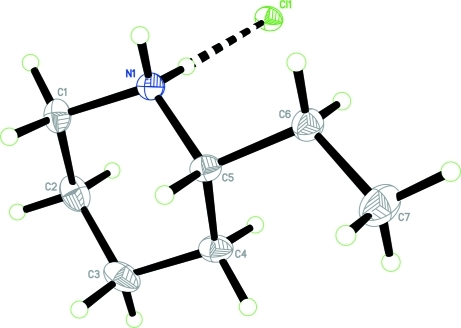
The asymmetric unit of the title compound, showing 50% probability displacement ellipsoids. An intermolecular N—H···Cl hydrogen bond is shown by a dashed line.

**Fig. 2.**
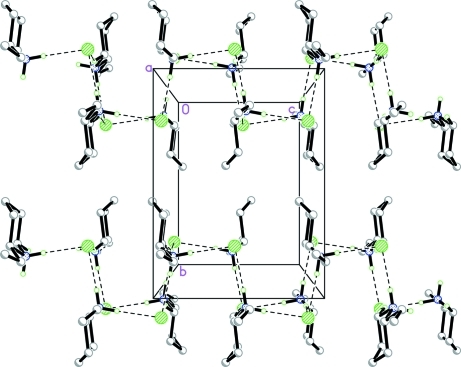
The crystal packing of the title compound, viewed along the a axis.

## supplementary crystallographic information

### Comment

Piperidine derivatives are the valued heterocyclic compounds in the field of medicinal chemistry. The piperidine nucleus is present in a wide range of biologically active compounds. For example, the binding properties of 4-diphenylacetoxy-*N*-methylpiperidine methiodide (4-DAMP) and its analogs have been evaluated at muscarinic receptors in human neuroblastoma NB-OK1 cells (M1 receptor subtype), rat heart (M2 subtype), rat pancreas (M3 subtype) and the putative M4 receptor subtype in striatum (Waelbroeck *et al.*, 1992). NMDA receptor antagonist properties of piperidine-2-carboxylic acid derivatives have also been reported (El Hadri *et al.*, 1995). Herein, we have present the crystal structure of the title compound (I).

The asymmetric unit of (I), (Fig. 1), consists of a 2-ethylpiperidinium cation and a chloride anion.  The piperidine (N1/C1–C5) ring adopts a chair conformation with puckering parameters Q = 0.5708 (9) Å, θ = 180.00 (9)° and φ = 282 (7)° (Cremer & Pople, 1975). In the crystal structure (Fig. 2), the cations and anions are connected by intermolecular N1—H1NA···Cl1, N1—H2NA···Cl1 and C1—H1A···Cl1 hydrogen bonds (Table 1), forming one-dimensional supramolecular chains along the *c*-axis.

### Experimental

In a round bottom flask, 25ml of tetrahydronfuran (THF) was mixed with 2-ethylpiperidine (0.01 mol, 0.8 g) with stirring.  Drops of benzylchloride (0.01 mol, 1.0 g) dissolved in THF was then added.  The reaction mixture was refluxed for 30 min.  The precipitate formed was washed with THF. The precipitate was then dissolved in methanol at room temperature. After few days, colourless needle-shaped crystals were formed by slow evaporation.

### Refinement

Atoms H1N1 and H2N1 were located from a difference Fourier maps and refined freely [N—H = 0.886 (13)–0.896 (14) Å]. The remaining H atoms were positioned geometrically (C—H = 0.98–1.00 Å) and were refined using a riding model, with *U*_iso_(H) = 1.2 or 1.5 *U*_eq_(C).  A rotating group model was applied to the methyl groups.

### Crystal data

**Table:**

C_7_H_16_N^+^·Cl^−^	*F*(000) = 656
*M**_r_* = 149.66	*D*_x_ = 1.157 Mg m^−^^3^
Orthorhombic, *P**b**c**n*	Mo *K*α radiation, λ = 0.71073 Å
Hall symbol: -P 2n 2ab	Cell parameters from 9898 reflections
*a* = 24.2052 (6) Å	θ = 2.3–36.9°
*b* = 9.7594 (3) Å	µ = 0.37 mm^−^^1^
*c* = 7.2764 (2) Å	*T* = 100 K
*V* = 1718.89 (8) Å^3^	Block, colourless
*Z* = 8	0.72 × 0.27 × 0.15 mm

### Data collection

**Table:**

Bruker SMART APEXII CCD area-detector diffractometer	4453 independent reflections
Radiation source: fine-focus sealed tube	3438 reflections with *I* > 2σ(*I*)
graphite	*R*_int_ = 0.045
φ and ω scans	θ_max_ = 37.3°, θ_min_ = 1.7°
Absorption correction: multi-scan (*SADABS*; Bruker, 2009)	*h* = −40→40
*T*_min_ = 0.778, *T*_max_ = 0.948	*k* = −16→16
50389 measured reflections	*l* = −12→12

### Refinement

**Table:**

Refinement on *F*^2^	Primary atom site location: structure-invariant direct methods
Least-squares matrix: full	Secondary atom site location: difference Fourier map
*R*[*F*^2^ > 2σ(*F*^2^)] = 0.040	Hydrogen site location: inferred from neighbouring sites
*wR*(*F*^2^) = 0.088	H atoms treated by a mixture of independent and constrained refinement
*S* = 1.07	*w* = 1/[σ^2^(*F*_o_^2^) + (0.0343*P*)^2^ + 0.4206*P*] where *P* = (*F*_o_^2^ + 2*F*_c_^2^)/3
4453 reflections	(Δ/σ)_max_ = 0.001
91 parameters	Δρ_max_ = 0.34 e Å^−^^3^
0 restraints	Δρ_min_ = −0.27 e Å^−^^3^

### Special details

**Table:**

Experimental. The crystal was placed in the cold stream of an Oxford Cryosystems Cobra open-flow nitrogen cryostat (Cosier & Glazer, 1986) operating at 100.0 (1) K.
Geometry. All s.u.'s (except the s.u. in the dihedral angle between two l.s. planes) are estimated using the full covariance matrix. The cell s.u.'s are taken into account individually in the estimation of s.u.'s in distances, angles and torsion angles; correlations between s.u.'s in cell parameters are only used when they are defined by crystal symmetry. An approximate (isotropic) treatment of cell s.u.'s is used for estimating s.u.'s involving l.s. planes.
Refinement. Refinement of F^2^ against ALL reflections. The weighted R-factor wR and goodness of fit S are based on F^2^, conventional R-factors R are based on F, with F set to zero for negative F^2^. The threshold expression of F^2^ > 2σ(F^2^) is used only for calculating R-factors(gt) etc. and is not relevant to the choice of reflections for refinement. R-factors based on F^2^ are statistically about twice as large as those based on F, and R- factors based on ALL data will be even larger.

### Fractional atomic coordinates and isotropic or equivalent isotropic displacement parameters (Å^2^)

**Table:**

	*x*	*y*	*z*	*U*_iso_*/*U*_eq_	
N1	0.39971 (3)	0.13082 (7)	0.95142 (9)	0.01471 (11)	
C1	0.45090 (3)	0.18923 (8)	1.03505 (12)	0.01927 (15)	
H1A	0.4838	0.1482	0.9761	0.023*	
H1B	0.4520	0.1670	1.1678	0.023*	
C2	0.45182 (4)	0.34373 (9)	1.00941 (12)	0.02298 (17)	
H2A	0.4543	0.3655	0.8768	0.028*	
H2B	0.4848	0.3823	1.0710	0.028*	
C3	0.39979 (4)	0.40873 (9)	1.08969 (12)	0.02384 (17)	
H3A	0.3999	0.5084	1.0647	0.029*	
H3B	0.3993	0.3955	1.2246	0.029*	
C4	0.34807 (4)	0.34393 (8)	1.00500 (12)	0.01959 (15)	
H4A	0.3148	0.3837	1.0638	0.024*	
H4B	0.3467	0.3664	0.8724	0.024*	
C5	0.34710 (3)	0.18860 (8)	1.02842 (10)	0.01526 (13)	
H5A	0.3452	0.1667	1.1625	0.018*	
C6	0.29921 (4)	0.11725 (8)	0.93081 (12)	0.01881 (14)	
H6A	0.3011	0.1382	0.7978	0.023*	
H6B	0.3033	0.0169	0.9455	0.023*	
C7	0.24270 (4)	0.16025 (11)	1.00354 (15)	0.02814 (19)	
H7A	0.2138	0.1106	0.9369	0.042*	
H7B	0.2378	0.2590	0.9855	0.042*	
H7C	0.2403	0.1388	1.1349	0.042*	
H1NA	0.4002 (5)	0.0410 (14)	0.9689 (17)	0.021 (3)*	
H2NA	0.4007 (4)	0.1476 (13)	0.830 (2)	0.024 (3)*	
Cl1	0.401145 (8)	0.182057 (18)	0.52892 (2)	0.01611 (5)	

### Atomic displacement parameters (Å^2^)

**Table:**

	*U*^11^	*U*^22^	*U*^33^	*U*^12^	*U*^13^	*U*^23^
N1	0.0164 (3)	0.0142 (3)	0.0135 (3)	0.0003 (2)	0.0004 (2)	0.0007 (2)
C1	0.0173 (3)	0.0207 (3)	0.0198 (3)	−0.0028 (3)	−0.0031 (3)	0.0026 (3)
C2	0.0263 (4)	0.0213 (4)	0.0213 (4)	−0.0086 (3)	−0.0037 (3)	0.0031 (3)
C3	0.0368 (5)	0.0158 (3)	0.0189 (3)	−0.0042 (3)	−0.0016 (3)	−0.0012 (3)
C4	0.0254 (4)	0.0137 (3)	0.0197 (3)	0.0020 (3)	0.0015 (3)	0.0000 (3)
C5	0.0176 (3)	0.0142 (3)	0.0140 (3)	0.0010 (2)	0.0026 (3)	0.0008 (2)
C6	0.0166 (3)	0.0188 (3)	0.0210 (3)	−0.0001 (3)	0.0004 (3)	0.0002 (3)
C7	0.0186 (4)	0.0286 (4)	0.0372 (5)	0.0007 (3)	0.0062 (4)	0.0015 (4)
Cl1	0.02022 (9)	0.01473 (8)	0.01339 (7)	−0.00044 (6)	0.00104 (6)	0.00007 (5)

### Geometric parameters (Å, °)

**Table:**

N1—C1	1.4935 (11)	C3—H3B	0.9900
N1—C5	1.5012 (10)	C4—C5	1.5256 (11)
N1—H1NA	0.886 (13)	C4—H4A	0.9900
N1—H2NA	0.896 (14)	C4—H4B	0.9900
C1—C2	1.5195 (12)	C5—C6	1.5275 (11)
C1—H1A	0.9900	C5—H5A	1.0000
C1—H1B	0.9900	C6—C7	1.5256 (13)
C2—C3	1.5264 (14)	C6—H6A	0.9900
C2—H2A	0.9900	C6—H6B	0.9900
C2—H2B	0.9900	C7—H7A	0.9800
C3—C4	1.5318 (13)	C7—H7B	0.9800
C3—H3A	0.9900	C7—H7C	0.9800
			
C1—N1—C5	114.10 (6)	C5—C4—C3	112.20 (7)
C1—N1—H1NA	108.0 (7)	C5—C4—H4A	109.2
C5—N1—H1NA	109.2 (7)	C3—C4—H4A	109.2
C1—N1—H2NA	108.0 (7)	C5—C4—H4B	109.2
C5—N1—H2NA	108.7 (7)	C3—C4—H4B	109.2
H1NA—N1—H2NA	108.7 (11)	H4A—C4—H4B	107.9
N1—C1—C2	109.93 (7)	N1—C5—C4	108.57 (7)
N1—C1—H1A	109.7	N1—C5—C6	107.40 (6)
C2—C1—H1A	109.7	C4—C5—C6	114.38 (7)
N1—C1—H1B	109.7	N1—C5—H5A	108.8
C2—C1—H1B	109.7	C4—C5—H5A	108.8
H1A—C1—H1B	108.2	C6—C5—H5A	108.8
C1—C2—C3	110.69 (7)	C7—C6—C5	113.19 (7)
C1—C2—H2A	109.5	C7—C6—H6A	108.9
C3—C2—H2A	109.5	C5—C6—H6A	108.9
C1—C2—H2B	109.5	C7—C6—H6B	108.9
C3—C2—H2B	109.5	C5—C6—H6B	108.9
H2A—C2—H2B	108.1	H6A—C6—H6B	107.8
C2—C3—C4	110.41 (7)	C6—C7—H7A	109.5
C2—C3—H3A	109.6	C6—C7—H7B	109.5
C4—C3—H3A	109.6	H7A—C7—H7B	109.5
C2—C3—H3B	109.6	C6—C7—H7C	109.5
C4—C3—H3B	109.6	H7A—C7—H7C	109.5
H3A—C3—H3B	108.1	H7B—C7—H7C	109.5
			
C5—N1—C1—C2	−58.09 (9)	C1—N1—C5—C6	−179.25 (6)
N1—C1—C2—C3	56.11 (9)	C3—C4—C5—N1	−54.61 (9)
C1—C2—C3—C4	−55.59 (9)	C3—C4—C5—C6	−174.51 (7)
C2—C3—C4—C5	55.68 (9)	N1—C5—C6—C7	176.69 (7)
C1—N1—C5—C4	56.59 (8)	C4—C5—C6—C7	−62.75 (10)

### Hydrogen-bond geometry (Å, °)

**Table:**

*D*—H···*A*	*D*—H	H···*A*	*D*···*A*	*D*—H···*A*
N1—H1NA···Cl1^i^	0.886 (14)	2.220 (14)	3.1054 (7)	176.9 (11)
N1—H2NA···Cl1	0.899 (15)	2.217 (15)	3.1149 (7)	177.8 (12)
C1—H1A···Cl1^ii^	0.99	2.80	3.6121 (8)	139

Symmetry codes: (i) *x*, −*y*, *z*+1/2; (ii) −*x*+1, *y*, −*z*+3/2.
